# Wearable in-ear pulse oximetry validly measures oxygen saturation between 70% and 100%: A prospective agreement study

**DOI:** 10.1177/20552076231211169

**Published:** 2023-11-07

**Authors:** Catherina AB Bubb, Michael Weber, Nadine Kretsch, Ralph Heim, Incinur Zellhuber, Sebastian Schmid, Simone M Kagerbauer, Johannes Kreuzer, Stefan J Schaller, Manfred Blobner, Bettina Jungwirth

**Affiliations:** 1 9184 Technical University of Munich, School of Medicine and Health, Department of Anaesthesiology and Intensiv Care Medicine, Munich, Germany; 2 Ulm University, Faculty of Medicine, Department of Anaesthesiology and Intensiv Care Medicine, Ulm, Germany; 3 Cosinuss° GmbH, Munich, Germany; 4 Charité – Universitätsmedizin Berlin, Corporate Member of Freie Universität Berlin and Humboldt Universitätzu Berlin, Department of Anesthesiology and Intensive Care Medicine (CCM/CVK), Berlin, Germany

**Keywords:** Telemetric monitoring, oxygen saturation, pulse rate, hypoxia, wearables, telemedicine

## Abstract

**Objectives:**

Postoperative monitoring outside intensive and post-anaesthesia care units is seldom, partly due to lack of suitable and approved systems. We therefore aim to validate the oxygen saturation (SpO_2_) and pulse rate measurement of the in-ear sensor c-med° alpha with a reference pulse oximeter.

**Methods:**

This prospective agreement study was conducted in 12 healthy (ASA 1) adult (18–50 years) volunteers according to the EN ISO 80601-2-61. The sitting volunteers were equipped with the finger pulse oximeter Rad-5 and two c-med° alpha sensors in each ear. The inspiratory oxygen content was reduced via a tight-fitting breathing mask to achieve five defined plateaus with stable SpO_2_ between 99% and 70%. The deviation of the SpO_2_ and pulse rate measurements of the c-med° alpha from those of the Rad-5 was calculated using the mean square error (A_rms_). Bias and limits of agreement between both devices were calculated using the Bland-Altman technique. The precision was compared based on the repeatability coefficients.

**Results:**

The c-med° alpha measured SpO_2_ had an A_rms_ = 1.9% relative to the Rad-5, a non-significant bias (−0.1% (-0.2% to 0.0%)), levels of agreement from −4.0% to 3.8%, and the same repeatability coefficient (0.8% vs. 0.8%). The c-med° alpha measured pulse rate did not deviate from the one measured with the certified finger pulse oximeter (bias: 0.1 min^−1^ (0 to 0.1 min^−1^), level of agreement: −3.6 to 3.7 min^−1^, A_rms_: 1.8 min^−1^).

**Conclusions:**

The c-med° alpha fulfils the EN ISO 80601-2-61 standard and is sufficiently accurate for measuring SpO_2_ and pulse rate in healthy adults at rest.

**Trial registration:**

EUDAMED No. CIV-21-03-036033

## Introduction

### Population of interest

The rate of surgical patients requiring intensified monitoring postoperatively are increasing in the developed countries.^1^ For many years, this was provided in post-anaesthesia or surgical intensive care units.^2^ However, an adaption of intensive care unit capacities to the increased requirements is very unlikely due to a lack of human and financial resources. Therefore, these patients are transferred early or directly to regular wards, where they are not adequately monitored potentially increasing the risk of ‘failure to rescue’.^3^ This is because the usual nursing supervision on normal wards is based on a four- to six-hour round,^4^ which is no longer practicable given the ratio of one nurse to 20 postoperative patients on many surgical wards, at least during the night.^5,6^ It is not surprising that unrecognised hypoxemia is a common postoperative complication.^7^ Importantly, postoperative complications account for 25% of all hospital mortality.^8^ On the other hand, a liberal transfer of postoperative patients to the intensive care unit, for instance for monitoring only, is associated with its own morbidity and mortality.^9^ Therefore and ‘Beyond “failure to rescue”: the time has come for continuous ward monitoring’.^10^

### Unmet needs

Conventional stationary vital monitoring is sometimes provided in specially reserved rooms in surgical wards. However, to monitor patients adequately in this setting, mobility must be severely restricted, which is known to delay recovery.^11^ The mobile telemetric monitoring known from regular cardiological wards is limited to heart rhythm analysis by means of an electrocardiogram. The most serious postoperative complications in surgical patients, however, are major bleeding, unrecognised hypoxaemia, delirium, and sepsis.^12^ To detect these at an early stage, it is essential to monitor pulse rate, oxygen saturation (SpO_2_), temperature, blood pressure, respiratory rate, and consciousness,^13^ while maintaining freedom of movement. While finger pulse oximetry is the most established non-invasive method for measuring pulse rate and SpO_2_, it has some relevant disadvantages: Firstly, measurements in critically ill patients are often compromised by peripheral hypoperfusion, e.g. in shock patients.^14^ Furthermore, in awake patients, movement artefacts can make the measurements unreliable. Finally, devices worn on the finger are cumbersome and uncomfortable in daily use, especially at night. Therefore, there is a need for a mobile device, a ‘wearable’, that not only has a high degree of validity, reproducibility, and precision in collecting vital signs, but is also convenient, lightweight, and non-obstructive.

### The investigational device

**Figure 1.**
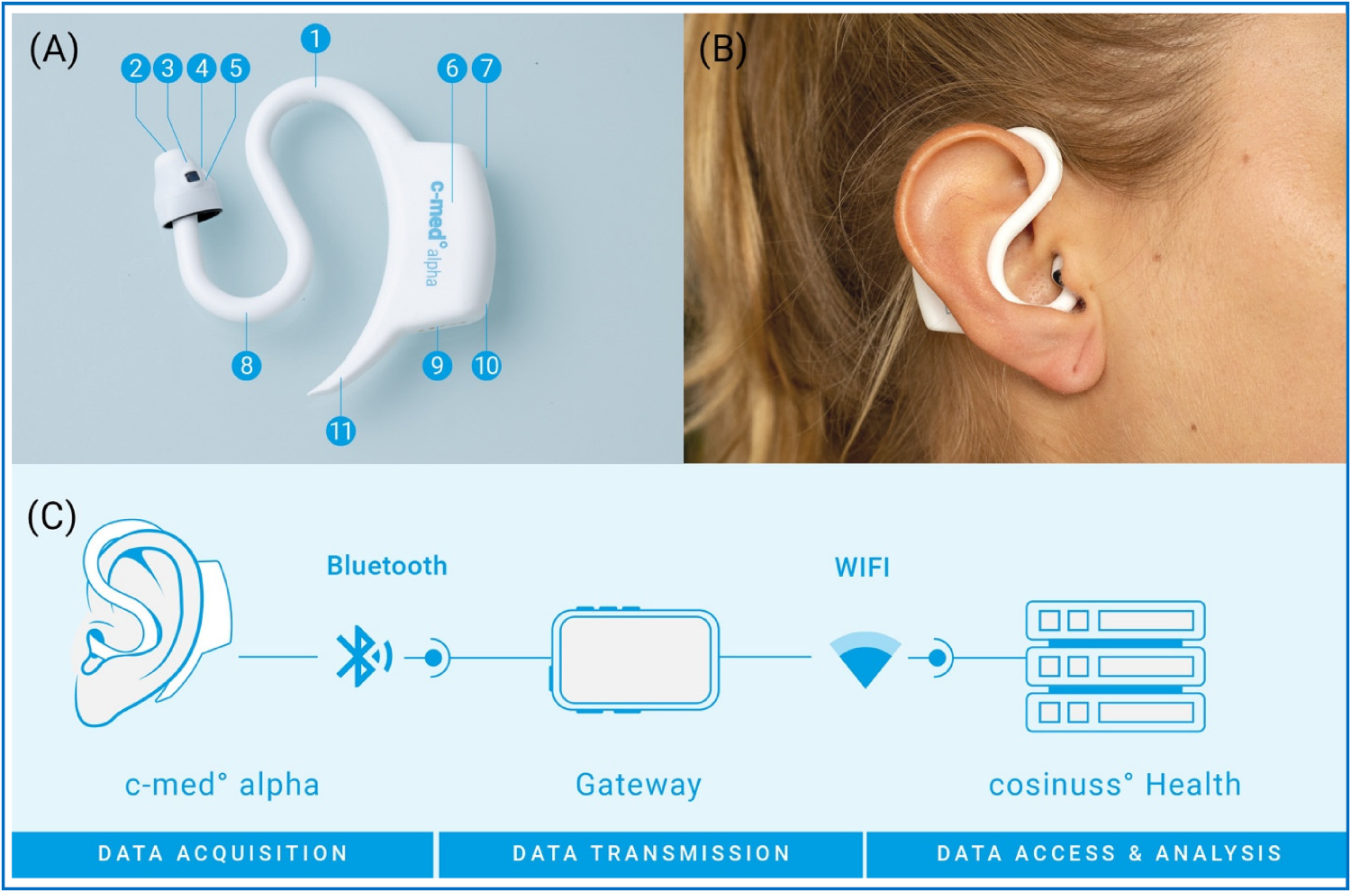
Description of the c-med alpha. (A) The in-ear sensor unit consists of the following components: (1) sensor neck, (2) infrared thermometer, (3) contact thermometer, (4) red/infrared light-emitting diode, (5) photo diode, (6) printed circuit board, battery and three-dimensional linear accelerometer, (7) status light-emitting diode, (8) anti-tragus curve, (9) charging contacts, (10) charging light-emitting diode, and (11) pickaxe. (B) A volunteer is wearing the c-med° alpha in her right ear. (C) Scheme of the data transfer from the in-ear sensor via the gateway to the server/interface using Bluetooth and network connection.

The c-med° alpha is a mobile, in-ear-wearable pulse oximeter for mobile use (Cosinuss GmbH, Munich, Germany). The in-ear sensor works on the basis of non-invasive photoplethysmography with two light-emitting diodes, one at 655 nm and the other at 940 nm wavelength, which are emitted alternatingly (Figure 1A-B).^15^ The emitted light intensity is controlled by the current applied to keep the reflected portion within the ideal measuring range of the sensor (20%–95%). The firmware calculates dynamically and beat-by-beat the SpO_2_ values from the medians of 20 consecutive red/infrared modulation ratios within a maximum of 30s. The c-med° alpha additionally is equipped with a three-dimensional linear accelerometer with an in-ear measuring range −157 to + 157 m/s^2^, a technical accuracy of 0.002 m/s^2^, and a variable sampling rate with standard 100 Hz via Bluetooth Low Energy (BLE 5.0). The c-med° alpha also has a contact thermometer and an infrared thermometer. The measured vital values are continuously transmitted from the c-med° alpha to any mobile device at a sampling rate of approximately 1 Hz, where the values are displayed on the c-med° mobile application (c-med° App).

### Aim of the study

The aim of the study was to validate the SpO_2_ and pulse rate measurement of the c-med° alpha in order to apply for the European medical CE certificate. The EN ISO 80601-2-61 standard for medical devices allows a non-invasive indirect method using a certified reference pulse oximeter with a known deviation from arterial blood gas analyses as the reference.^16^ The agreement must be tested at five predefined saturation plateaus between 99% and 70%. Therefore, we compared the SpO_2_ measurements of the c-med° alpha with those of the reference pulse oximeter Rad-5 (Masimo, Irvine, California, USA) in healthy adult volunteers at rest under normobaric hypoxaemia.

## Methods

### Study characteristics

This prospective agreement study was conducted at the university hospital Klinikum rechts der Isar of the School of Medicine at Technical University of Munich in Munich, Germany. The study is reported according to the GRRAS recommendations.^17^ The trial was conducted in accordance with the principles of the Declaration of Helsinki and Good Clinical Research guidelines. The study was prospectively registered at the German DIMDI register (application number 00013336) and the EUDAMED register (No. CIV-21-03-036033).

### Volunteers

After positive approval by the ethics committee (679/21 Mf-KK) and the regulatory authority, the subjects were screened for eligibility, and written informed consent was requested. According to the DIN EN ISO 80601-2-61:2019,^16^ inclusion criteria are healthy (ASA 1) adults (18–50 years), non-smoking or smoke-free for 48 hours, including at least 25% participants of each sex. Due to a possible influence of skin pigmentation on the optical measurement of saturation,^18^ 15% of the subjects had to have darker skin according to the Fitzpatrick scale defined by Type IV, V, and VI.^19^ Exclusion criteria were pregnancy or breastfeeding.

### Study protocol

After the medical examination, the volunteers were positioned sitting in a chair with the option of putting their legs up for increased comfort. For the volunteers’ safety, vital signs including ECG, arterial pressure, and SpO_2_ were monitored continuously during the study using a routine anaesthesia monitoring device (Carescape B650 patient monitor, GE Healthcare, Helsinki, Finland). Since hypoxia can lead to impaired consciousness, we included processed electroencephalographic monitoring in addition to direct clinical observation for its early detection (E-Entropy Module, GE Healthcare, Helsinki, Finland). Then, the investigational devices were attached: the reference pulse oximeter Rad-5 (finger clip LNCS DCI, Masimo, Irvine, California, USA – emitting lights of 660 nm and 905 nm, display of mean values of the last four consecutive seconds) and two c-med° alpha sensors, one in each ear. Finally, the measuring systems including the interfaces for data recording were checked for proper function.

After the volunteers had become accustomed to the set-up, a breathing mask with a non-rebreathing valve was placed firmly over the nose and mouth, and they were instructed to breathe at a rate of approximately ten per minute. To support a stress-free and regular breathing, the volunteers were shown a video with a breathing volume curve in a continuous loop. After a five minute baseline period, an altitude generator (Everest Summit II Hypoxic Generator, Gairit©, Salzburg, Austria) was connected to the breathing mask via a tube with a respiratory reservoir. The altitude generator reduces the inspiratory oxygen content by generating nitrogen from the room air and adding it to the inhaled air. Starting from the room air, the gradual desaturation of the volunteers was achieved by increasing the fraction of nitrogen to the inspiratory air. To achieve five defined ranges with stable SpO_2_ (96–99%, 90–95%, 84–89%, 77–83%, and 70–76%), the nitrogen inflow was adapted according to the requirements of each volunteer.

The duration of each desaturation trial including all ranges was limited to 25 min. If some volunteers did not reach all five ranges, either due to hyperventilation or other reasons, only a second attempt to reach all ranges was allowed. After the measurements, all subjects remained at the site until possible side effects could be ruled out.

### Data collection

From the c-med° alpha and the cosinuss° gateway, data was automatically sent via an internet connection to the cosinuss° Health platform for analysis and archiving (Figure 1C). The SpO_2_ and pulse rate values of the Masimo Rad-5 pulse oximeter were recorded with a camera connected to the cosinuss° gateway and also uploaded to the cosinuss° Health platform to obtain a synchronised continuous reference measurement. The sampling rate of the c-med° alpha and the frame rate of the camera were both 1 Hz.

The data obtained during the screening and clinical examination of the volunteers are documented in the case report forms and transferred to the cosinuss° Health platform after pseudonymisation. The database is closed for further data entry and accessible for the study team.

### Data management

**Figure 2.**
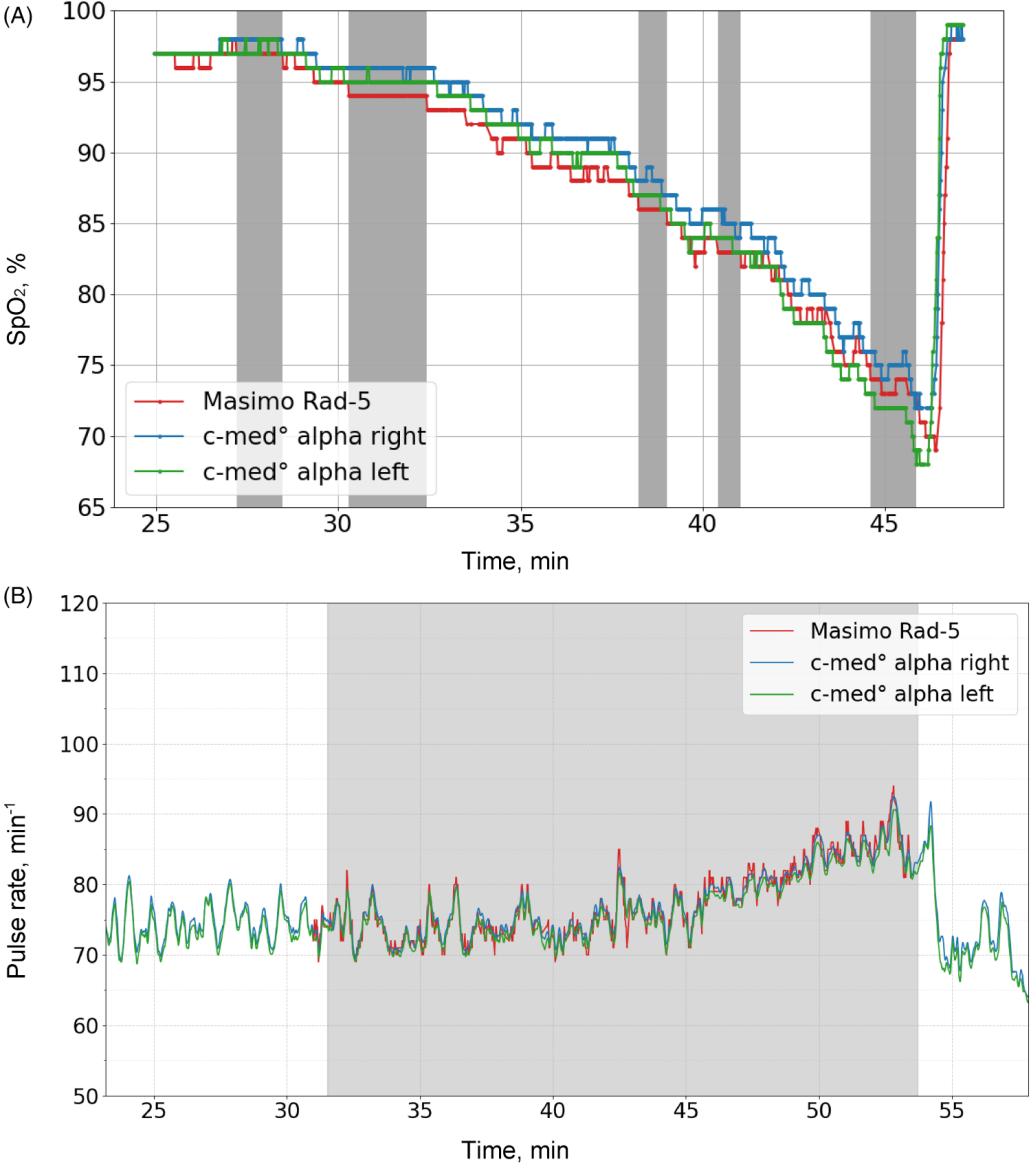
Example plots. Values of the Masimo Rad-5 are shown in red, those of the c-med° alpha in green (right ear) or blue (left ear). The grey-coloured areas highlight the values used for analyses. (A) SpO_2_ measurements with highlighted stable plateaus of consecutive SpO_2_ values measured with the Rad-5 and differing by no more than 2% over a period of at least 30s. (B) Pulse rate measurement with the time interval of the desaturation measurement.

The data were analysed according to the EN ISO standard method at the five defined ranges with stable SpO_2_.^16^ A plateau is defined as consecutive SpO_2_ values within a given range differing by ≤ 2% during a period of at least 30s as measured by the Rad-5. If more suitable plateaus were found within a SpO_2_ range, that with the least variation in SpO_2_ values and longest duration was chosen. The two measurement sites (the left and the right ear) generated two data sets for each plateau. For each volunteer, the data of both measurement sites were included into the final data set. An example of how the plateaus were decided is presented in Figure 2A.

The data pairs within the plateaus were further reduced for statistical analysis. First, SpO_2_ signals were resampled at a rate of one value per second. If multiple values occurred within a second, the median was used. The analysed data pairs must be evenly distributed over the five predefined ranges. Therefore, only data pairs of volunteers who reached all ranges and a maximum of ten pairs from the middle of each plateau were used for analysis. In addition, equal weighting was applied to all plateaus and subjects.

We determined the accuracy of the c-med° alpha measured pulse rate also using the Masimo Rad-5 device. For comparison, we used pairs of the median pulse rate of both devices at ten second intervals during the desaturation manoeuvre (an example is given in Figure 2B).

In addition to the planned analyses of the data from the stable SpO_2_ plateaus, sensitivity analyses were carried out for all data, i.e. including those outside the stable SpO_2_ plateaus.

### Sample size calculation

In order to meet the requirements of the international standards for oximeter devices, the Bland-Altman method was used.^20^ To achieve sufficient power, Bland and Altman recommend 100 data pairs and, in the case of repeated (dependent) measurements, further 20 pairs. The data pairs must come from a sufficiently large number of different subjects, i.e. the number of subjects must be greater than the number of repeated measurements. In this study, measurements were recorded at five different saturation plateaus (five dependent measurements). With 12 volunteers, five plateaus, and two data pairs of each plateau, we met the required power (12 × 5 × 2 = 120 data pairs). Assuming that half of the subjects do not reach all five plateaus even after a second attempt, a maximum of 24 volunteers were scheduled for screening. It was specified that the study would be terminated (a) if all five plateaus (with at least two pairs of data per plateau) were reached in 12 subjects and (b) after 10 subjects, if more than half of the subjects did not reach all plateaus once, after two attempts.

### Statistical analysis

Statistical analyses were performed using Python 3 (Python Software Foundation).^21^ SpO_2_ measured with the c-med° alpha (SpO_2,c__-med_) was related to the values measured with reference device Rad-5 (SpO_2,Rad__-5_). The accuracy was calculated using the root mean square error method (A_rms_):

$$A_{rms,trial}=\;\sqrt{\frac{\sum_{i=1}^{n}\;{\left(SpO_{2,c-med}\;-SpO_{2,Rad-5}\right)}^{2}}{n}}$$

The accuracy of SpO_2_ measured with the c-med° alpha relative to that measured with arterial blood gas analyses (A_rms,c-med_) is the composition of the A_rms,trial_ relative to the Rad-5 and the accuracy of the Rad-5 relative to arterial blood gas analyses (A_rms,Rad-5_):

$$A_{rms,\;c-med}=\;\sqrt{A_{rms,trial}^{2}+\;A_{rms,\;Rad-5}^{2}}$$

According to the user manual, the Masimo Rad-5 has an accuracy A_rms,Rad-5_ ≤ 2% to measure SpO_2_ compared with arterial blood gas analyses.^22^ A sufficient accuracy of the c-med° alpha can be assumed if the A_rms,c-med_ ≤ 4% under resting conditions in the range of 70–100% SpO_2_.^16^

In addition, agreement was analysed calculating bias and the limits of agreement (LoA) between values of the c-med° alpha and the Masimo Rad-5, as suggested by Bland-Altman.^20,23^ The bias (*D̅*) was defined as the mean difference between c-med° alpha and Rad-5. The corresponding *LoA* were defined as

$$LoA=\bar{D}\pm 1.96\;{\hat{\sigma }}_{D}^{}$$

where ${\hat{\sigma }}_{D}^{2}$ was estimated by

$${\hat{\sigma }}_{D}^{2}={\hat{\sigma }}_{\bar{D}}^{2}+\frac{{\hat{\sigma }}_{w_{c-med}}^{2}}{2}+\frac{{\hat{\sigma }}_{w_{RAD-5}}^{2}}{2}$$

The precision of both devices was compared based on their 95% repeatability coefficient *r* defined as 1.96 times the standard deviation of the differences between two consecutive measurements, i.e. $r=1.96\;\sqrt{2}\;{\hat{\sigma }}_{w}$ , where ${\hat{\sigma }}_{w}$ was estimated by the square root of the residual mean square. The 95% confidence interval (CI) for the repeatability coefficient was constructed using the sample variance of *r*, $\hat{Var(r)}\;=\frac{1.96^{2}{\hat{\sigma }}_{w}^{2}}{n}$ , where *n* is the number of comparisons. The repeatability coefficient defines the 95% tolerance interval around a measured value in which a repeat measurement on the same subject acquired under identical conditions should fall within 95% probability. Repeatability coefficients for the c-med° alpha and the Rad-5 SpO_2_ measurement were compared using F-tests. The 95% CI for the repeatability coefficient *r*, bias, and the LOA were constructed using the Python scipy.stats package.

The accuracy, agreement, and repeatability of the mean pulse rate of ten second-intervals measured with c-med° alpha compared to that measured with the Rad-5 was analysed analogously to SpO_2_. The accuracy of the pulse rate measurement was calculated by the root mean square error from the pulse rate pairs of the study data, combined with the accuracy of the Rad-5 given in its user manual with three per minute relative to a Biotek Index 2 simulator.^22^ An acceptable combined accuracy of the c-med° alpha to measure pulse rate was predefined to five per minute.

Biometric and demographic data are given as means ± standard deviation.

## Results

### Biometrics

Fifteen adults underwent the screening process. Two volunteers did not meet the inclusion criteria, one due to a suspected Wolff-Parkinson-White syndrome and the other for organisational reasons. Hence, 13 volunteers were included in the study. One volunteer was excluded during the study because she felt uncomfortable during the desaturation test. Twelve volunteers completed the study and, data from all, totalling 1200 data points, were included in further data analysis. No serious adverse event occurred. The mean age was 28 ± 6 years, the mean BMI was 23 ± 2 kg/m^2^, and four out of 12 volunteers were female. The skin type distribution according to the Fitzpatrick scale was one type I, five type II, three type III, two type IV, and one type V (Table S1). Eight of the 12 volunteers reached all five desaturation plateaus on the first trial, and four volunteers needed a second one due to exceeding the time limit of 25 min. The mean total time for the first trial was 20 ± 3 min and for the second trial 18 ± 2 min. The inspiratory oxygen fraction during the five plateaus was 18.3% ± 1.7%, 15.0% ± 1.7%, 11.9 ± 1.3, 10.3% ± 0.9%, and 9.1% ± 0.7%, respectively.

### Oxygen saturation

**Figure 3.**
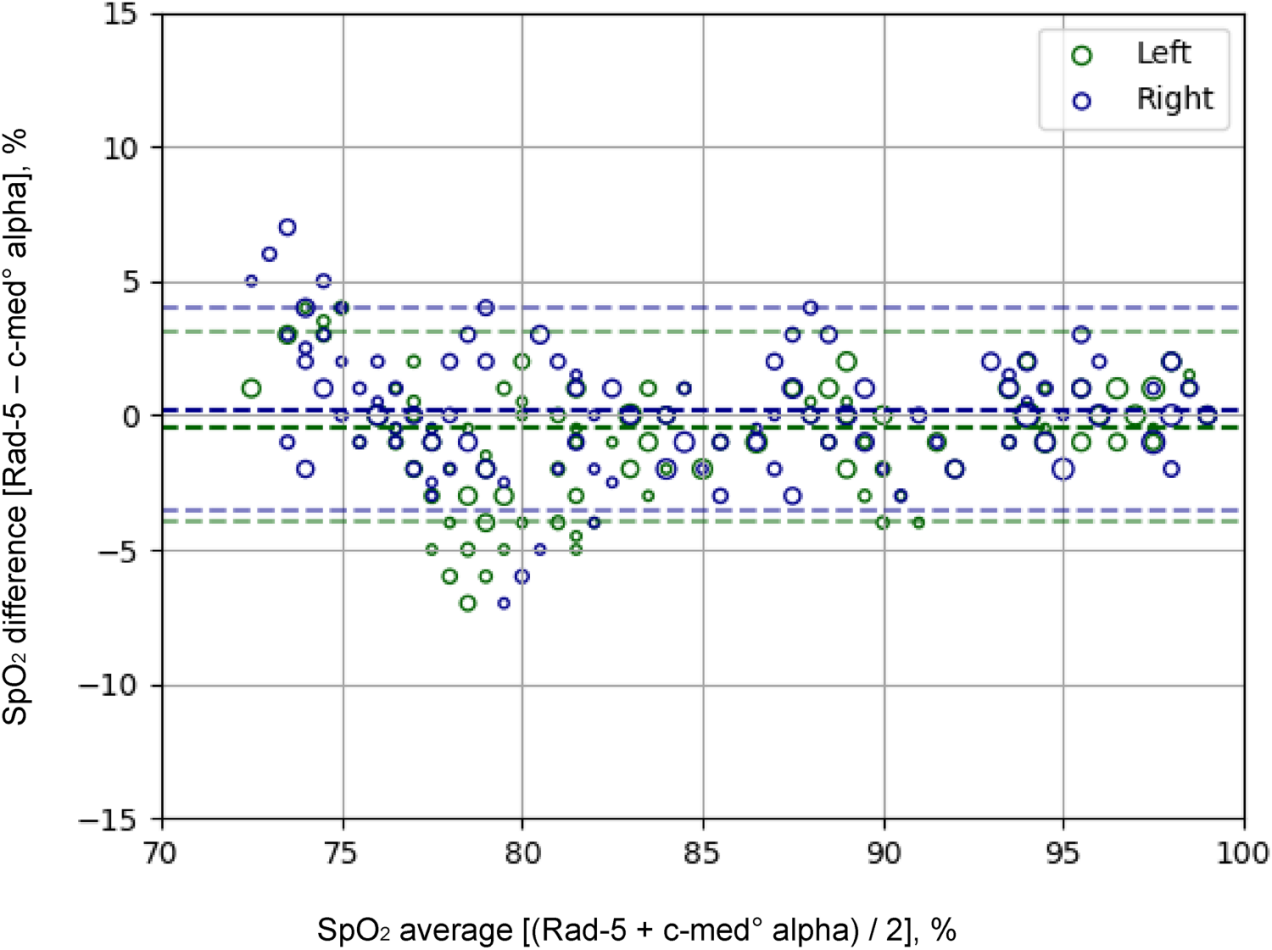
Bland-Altman analysis of the Rad-5 and the c-med alpha° of the SpO_2_ at five desaturation plateaus. Differences between simultaneous SpO_2_ readings of the c-med° alpha in-ear oximetry and the finger pulse oximeter (Masimo Rad-5). In-ear values were taken in both ears; the SpO_2_ pairs of the fingers with the left ear are green, those with the right ear are blue. The thick dashed line indicates the mean difference of the measurements (bias), the thin dashed lines the 95% limits of agreement. The size of markers is proportional to the number of readings.

The c-med° alpha measured SpO_2_ had an accuracy of A_rms,trial_ = 1.9% relative to the one measured with Rad-5 resulting in an accuracy relative to arterial blood gas analyses of A_rms,c__-med_ = 2.9%. On all SpO_2_ plateaus of > 76%, the A_rms,trial_ was < 2.0% but reached 3.0% at the plateau with the lowest SpO_2_ values (Table 1). The mean difference of the SpO_2_ measurements was −0.1% (-0.2% to 0.0%), indicating that there was no significant bias between both devices. The LOA (95% of all measured values at the stable plateaus) were between −4% and 4%, the maximum differences were −7% and +7%, which were observed at SpO_2_ values ≤ 80% only. (Table 1 and Figure 3). Sensitivity analyses also including the values outside the stable plateaus revealed a bias of 0.2% (0.2% to 0.3%) LOA between −5% and +5% (Supplementary Figure S1).

**Table 1.** Agreement between oxygen saturation (SpO_2_) measured in-ear (c-med° alpha) and on the finger (Rad-5).

SpO_2_ Plateau, %	Bias (95% CI), %	Lower LOA (95% CI), %	Upper LOA (95% CI), %	A_rms_
99–96*	0.4 (0.3 to 0.5)	-1.6 (-1.8 to -1.5)	2.4 (2.3 to 2.6)	1.1
95–90*	-0.1 (-0.2 to 0.1)	-2.4 (-2.6 to -2.2)	2.2 (2.0 to 2.4)	1.2
89–84*	-0.4 (-0.6 to -0.2)	-3.6 (-3.9 to -3.4)	2.8 (2.5 to 3.1)	1.7
83–77*	-0.3 (-0.5 to -0.1)	-3.9 (-4.3 to -3.6)	3.3 (3.0 to 3.7)	1.9
76–70*	-0.2 (-0.5 to 0.2)	-6.1 (-6.7 to -5.6)	5.8 (5.3 to 6.4)	3.0
All plateaus*	-0.1 (-0.2 to 0.0)	-3.8 (-4.0 to -3.7)	3.6 (3.5 to 3.8)	1.9
Left ear	-0.4 (-0.6 to -0.3)	-4.0 (-4.2 to -3.8)	3.1 (2.9 to 3.3)	1.9
Right ear	0.2 (0.1 to 0.4)	-3.6 (-3.8 to -3.4)	4.0 (3.8 to 4.2)	1.9

*Note.* To illustrate the small 95% CIs, one digit after the decimal point is given, although the SpO_2_ is only measured as an integer.

*both ears.

CI = confidence interval, LoA = limit of agreement, A_rms_ = accuracy using root mean square error.

The repeatability coefficients were < 1% and did not differ between both devices at the stable SpO_2_ plateaus (*p* = 0.4; Table 2). At both SpO_2_ plateaus of ≥ 90%, the c-med° alpha had significantly higher repeatability coefficients, but at two of three SpO_2_ < 90%, they were lower. Sensitivity analyses that also included the values outside the stable plateaus resulted in significantly (*p* < 0.001) lower repeatability coefficients for c-med° alpha 1.2% (1.2% to 1.2%) compared to those for Rad-5 1.3% (1.3% to 1.4%).

**Table 2.** Precision of oxygen saturation (SpO_2_) measured in-ear (c-med° alpha) and on the finger (Rad-5) given as repeatability coefficient.

	Repeatability Coefficient (95% CI), %		
SpO_2_ Plateau, %	c-med° Alpha	Rad-5	*p*-value
99–96*	0.6 (0.5 to 0.6)	0.3 (0.2 to 0.3)	< 0.001
95–90*	0.7 (0.7 to 0.8)	0.4 (0.4 to 0.5)	< 0.001
89–84*	0.8 (0.7 to 0.9)	1.1 (1.0 to 1.3)	< 0.001
83–77*	0.6 (0.6 to 0.7)	0.8 (0.7 to 0.9)	0.001
76–70*	1.1 (1.0 to 1.2)	1.0 (0.9 to 1.1)	0.048
All plateaus*	0.8 (0.8 to 0.9)	0.8 (0.8 to 0.9)	0.4
Left ear	0.8 (0.8 to 0.9)	0.8 (0.8 to 0.9)	0.4
Right ear	0.8 (0.8 to 0.9)	0.8 (0.8 to 0.9)	0.5

*Note.* *both ears. CI = confidence interval.

To illustrate the small 95% CIs, one digit after the decimal point is given, although the SpO_2_ is only measured as an integer.

No significant differences were found when the data samples were divided into groups of Fitzpatrick scales (I–III or IV–VI) or different genders (male or female).

### Pulse rate

**Figure 4.**
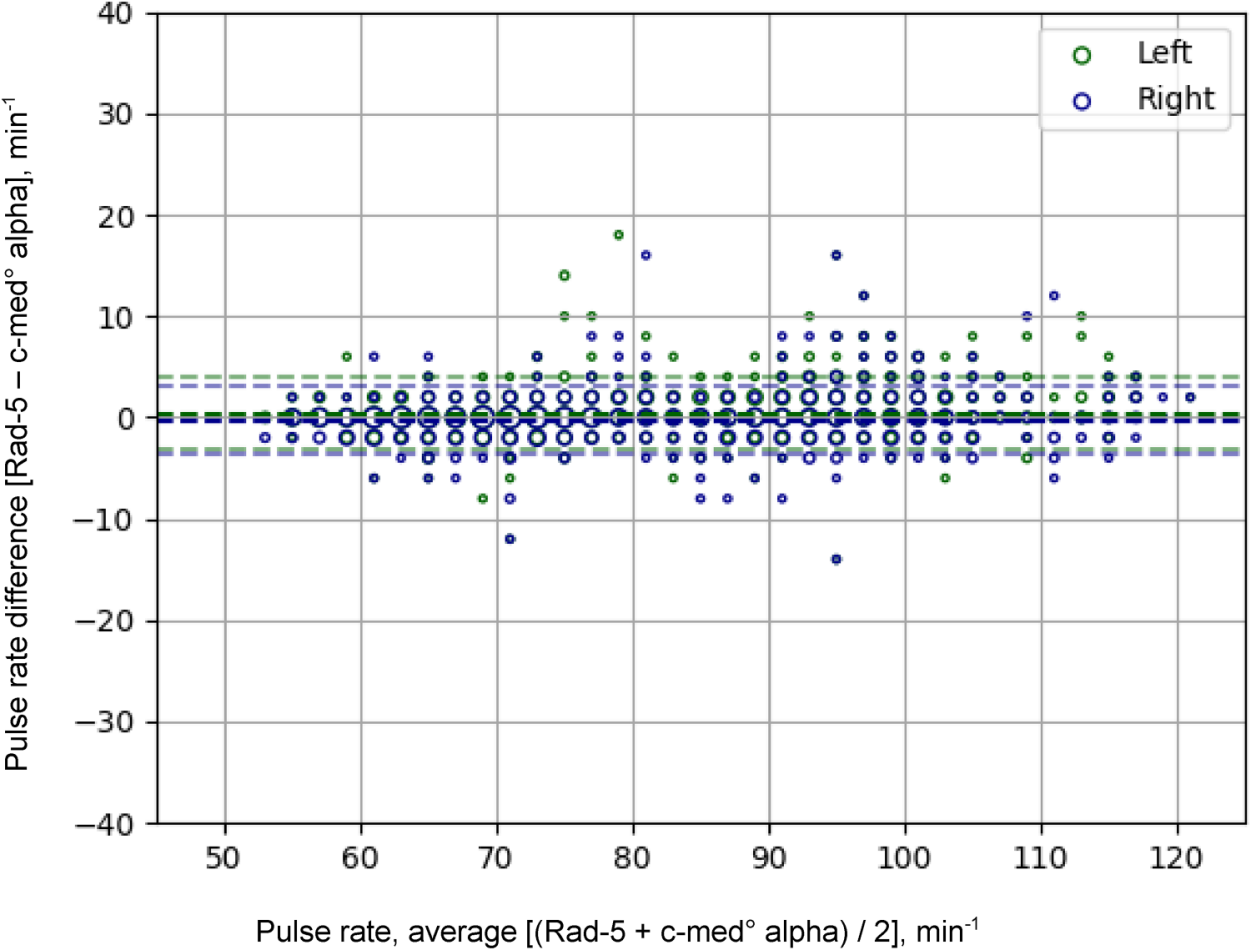
Bland-Altman analysis of the Rad-5 and the c-med alpha° of the pulse rate. Differences between simultaneous pulse rate readings of the c-med° alpha and the finger pulse oximeter (Masimo Rad-5) during desaturation exercise. In-ear values were taken in both ears; the pulse rate pairs of the fingers with the left ear are green, those with the right ear are blue. The thick dashed line indicates the mean difference of the measurements (bias), the thin dashed lines the 95% limits of agreement. The size of markers is proportional to the number of readings.

The c-med° alpha measured pulse rate had an accuracy of A_rms,trial_ = 1.8 min^−1^ relative to the one measured with the Rad-5 resulting in an accuracy of A_rms, c__-med_ = 3.5 min^−1^ relative to standardised pulse simulator. At higher pulse rates, the A_rms,trial_ increased with a maximum of 3.1 min^−1^ for tachycardia > 100 min^−1^ (Table 3). The mean difference of the pulse rate measurements was 0.1 min^−1^ (–3.5 to 3.6 min^−1^) indicating no statistically significant bias between both devices. The LOA (95% of all measured values) were between −4 min^−1^ and 4 min^−1^, the maximum differences were −7 min^−1^ and 5 min^−1^ (Figure 4 and Table 3).

**Table 3.** Agreement between pulse rate (PR) measured in-ear (c-med° alpha) and on the finger (Rad-5).

PR ranges, min^−1^	Bias (95% CI), min^−1^	Lower LOA (95% CI), min^−1^	Upper LOA (95% CI), min^−1^	A_rms_
≤ 60*	−0.4 (−0.6 to −0.3)	−2.2 (−2.3 to −2.1)	1.3 (1.2 to 1.5)	1.0
60–80*	−0.2 (−0.3 to −0.2)	−2.7 (−2.7 to −2.6)	2.2 (2.1 to 2.3)	1.3
80–100*	0.3 (0.1 to 0.4)	−4.0 (−4.2 to −3.9)	4.6 (4.4 to 4.7)	2.2
> 100*	1.2 (0.9 to 1.5)	−4.4 (−4.8 to −4.0)	6.8 (6.5 to 7.3)	3.1
All values*	0.0 (−0.1 to 0.1)	−3.5 (−3.6 to −3.4)	3.6 (3.5 to 3.7)	1.8
Left ear	0.3 (0.2 to 0.4)	−3.3 (−3.4 to −3.1)	3.9 (3.8 to 4.0)	1.9
Right ear	−0.3 (−0.3 to −0.2)	−3.7 (−3.8 to −3.6)	3.2 (3.1 to 3.3)	1.8

*Both ears. CI = confidence interval, LoA = limit of agreement, A_rms_ = accuracy using root mean square error.

To illustrate the small 95% CIs, one digit after the decimal point is given, although the PR is only measured as an integer.

### Data transfer

In total, approximately 100.000 data points for SpO_2_, pulse rate, acceleration, and temperature were recorded over a period of approximately 15 hours and successfully transferred to the cosinuss° Health platform.

## Discussion

To the best of our knowledge, the c-med° alpha is the first in-ear pulse oximeter for which the medical standard for SpO_2_ measurement could be proven by a low deviation from blood gas analyses in an indirect comparison (A_rms_ = 2.9%). Thus, the c-med° alpha fulfilled the regulatory requirements set by the EN ISO 80601-2-61 and has accordingly received the European medical CE certificate based on the results of this study.^16^ In direct comparison with a reference finger pulse oximeter, it confirmed the high quality of the SpO_2_ measurement by a non-significant bias (−0.1% (−0.2% to 0.0%)), a narrow range between the levels of agreement (−4.0% to 3.8%) and a comparably good precision (repeatability coefficient: 0.8% vs. 0.8%). In addition, the c-med° alpha measured pulse rate did not deviate from the one measured with the certified finger pulse oximeter (bias: 0.1 min^−1^ (0 to 0.1 min^−1^), level of agreement: −3.6 to 3.7 min^−1^, A_rms_: 1.8 min^−1^). Furthermore, the study results demonstrated reliable data transmission via the cosinuss° interface.

### The methods and its implications

There are two methods for the clinical validation of oxygen saturation measurements according to the EN ISO 80601-2-61 standards for medical pulse oximeters^16^: an invasive method using arterial blood gas analyses as reference^24^ and the non-invasive indirect method, which we have applied in this study. Accordingly, we incorporated the deviation of the Rad-5 from arterial blood gas analyses into the total error of the c-med° alpha.^16^ The non-invasive approach had the advantage of reducing the burden on the volunteers, avoiding sampling errors, but most importantly comparing the precision of the SpO_2_ and pulse rate measurement of both devices by their repeatability coefficients, which requires continuous measurements.

The FDA Guidance for Pulse Oximeters differs from EN ISO 80601-2-61 in the slightly smaller accepted deviation from the reference of 3.5% and in the recommendation of the invasive method whenever methodologically possible.^19^ Although the deviation of the SpO_2_ of the c-med° alpha calculated with the non-invasive method is below 3.5%, the omission of additional sampling of arterial blood during plateaus is therefore a limitation of our study.

Since there is neither a reference system nor a technical standard defining requirements on pulse rate measurement accuracy, we determined the accuracy of the c-med° alpha measured pulse rate also comparing it with the Masimo Rad-5 device during the normobaric hypoxia exercise.

Recently, some wearable wrist oximeters, such as the Apple Watch 6, the Garmin Fēnix 6, and the Oxitone 1000M, have also been studied under normobaric hypoxia.^25–27^ In each study, only very few pathological SpO_2_ values < 90% were included in the evaluation, in no case values < 80%. Since the European medical CE certification requires accuracy of SpO_2_ measurements over five ranges of stable SpO_2_, three of which must be pathological and one of which must approximate 70%, these studies provide only limited information about their usability for clinical use. Although not published in a scientific journal, the study report on the FDA-approved Oxitone 1000M showed an agreement with a reference CO-oximetry between 70% and 100%.^28^ We achieved the pathological SpO_2_ ranges 84–89%, 77–83%, and 70–76% with our comprehensive study design.

There is no evidence that the skin in the external auditory canal is pigmented differently from the skin on the back of the fingers.^29^ Accordingly, the SpO_2_ values of volunteers with higher skin pigmentation did not differ more between the two methods in our study (Table S1 and S2). In addition, the c-med° alpha controls the light intensity emitted by the light-emitting diode. When the measured light yield falls below 20%, e.g. because of adsorption by high melanin content of the skin,^30^ the current applied to the diode is increased to achieve a yield of 50%.

Several studies have shown that the SpO_2_ can be measured most accurately at rest, and movement degrades the accuracy significantly.^31–33^ Although there are pulse oximeters in the market, including the reference device used in this study, which claim to provide valid SpO_2_ measurements under motion, they usually describe very specific, minor movements (i.e. rubbing and tapping) that are not relevant for in-ear measurements and far from patients walking around. In this study, we validated the SpO_2_ and pulse rate values of the c-med° alpha at rest and at the SpO_2_ plateaus defined by the reference device. Therefore, we did not see any motion artefacts and consequently cannot describe their possible effects on the accuracy of the SpO_2_ and pulse rate measurements of the c-med° alpha. The results of studies focusing on motion disturbances in the measurement quality of wearable wrist oximeters^27,34,35^ and preclinical experience with the c-med° alpha^36^ suggest that movement artefacts will be an important topic for future clinical research and technical development.

### Expected benefits of the wearable system in the population of interest

Providing continuous monitoring of vital signs, the c-med° alpha offers prolonged monitoring of postoperative, high-risk patients on the surgical ward while maintaining their mobility. By integrating data collection in the hospital central monitoring system via Bluetooth, it offers a low-effort technical implementation making it a better alternative to established monitoring devices based on telemetry. In addition, the c-med° alpha is a robust, disinfectable, therefore reusable, and cost-effective device. Similar to wrist oximeters and unlike conventional portable monitoring, it exploits the daily habits of many patients who are used to wearing headphones or hearing aids to achieve high compliance.

A high frequency of false alarms and the associated risk of alarm fatigue would not be helpful in achieving the goal of timely detection of possible complications.^37^ Therefore, it is not yet clear how to deal with the expected instability of the measurements by any wearable, especially in mobile use,^31^ and how the values have to be interpreted.

The promising experience with early warning scores puts the importance of individual pathological values into perspective and could pave the way to solving the problem.^38^ At least in early but still treatment-emergent stages, it makes more sense to pay attention to several symptoms of a complication rather than focusing on one. Wearables can have a significant advantage in this respect due to their simultaneous and continuous recording of several parameters. The c-med° alpha, for instance, directly measures SpO_2_, pulse rate,^39^ temperature,^40^ and acceleration^41^ and indirectly provides estimates of blood pressure,^42,43^ respiratory rate,^36,40^ and probably consciousness from patterns of these measured signals, i.e. the c-med° alpha is able to provide information on all six parameters of the ‘National Early Warning Score 2’.^13^ Development of alarm algorithms based on the trends of several parameters followed by their clinical evaluation may increase the positive as well as negative predictive values of their alarms.

### Open gaps

Our sensitivity analyses, which include the SpO_2_ pairs outside the stable plateaus, show that the agreement between the devices and the precision of each device suffers with changing SpO_2_ values. The averaging of c-med° alpha by medians of 20 consecutive values within a maximum of 30s aims at a high robustness against artefacts in planned mobile deployment. However, the clinical value of the c-med° alpha must still be investigated especially under changing SpO_2_ values and different types of movement. Although the study protocol meets the regulatory requirements with the inclusion of three volunteers with Fitzpatrick ≥ IV, c-med° alpha has yet to be studied in subjects with skin pigmentation of the highest Fitzpatrick scales VI. Furthermore, stability and accuracy of data collection has yet to be proven in the clinical setting. Another open gap is the lack of clinical studies demonstrating the technology concept and integration in a hospital ward. Finally, the wearing comfort of the c-med° alpha was not assessed in our study, which is crucial especially for long-term use, and a patient referral may be needed for correct use.

## Conclusion

Our study demonstrates that the c-med° alpha accurately measures blood oxygen saturation and pulse rate in healthy adults under normobaric hypoxia at rest, thus meeting the requirements of the ISO 80601-2-61 standard for a medical device.

## Supplemental Material

sj-docx-1-dhj-10.1177_20552076231211169 - Supplemental material for Wearable in-ear pulse oximetry validly measures oxygen saturation between 70% and 100%: A prospective agreement study Click here for additional data file. Supplemental material, sj-docx-1-dhj-10.1177_20552076231211169 for Wearable in-ear pulse oximetry validly measures oxygen saturation between 70% and 100%: A prospective agreement study by Catherina AB Bubb, Michael Weber, Nadine Kretsch, Ralph Heim, Incinur Zellhuber, Sebastian Schmid, Simone M Kagerbauer, Johannes Kreuzer, Stefan J Schaller, Manfred Blobner and Bettina Jungwirth in DIGITAL HEALTH
