# Supplementary material for: Wearable in-ear pulse oximetry validly measures oxygen saturation between 70% and 100%: A prospective agreement study
Source: Digit Health. 2023 Nov 7;9:20552076231211169. doi: 10.1177/20552076231211169 (PMC10631342; doi:10.1177/20552076231211169)
Supplement: sj-docx-1-dhj-10.1177_20552076231211169 - Supplemental material for Wearable in-ear pulse oximetry validly measures oxygen saturation between 70% and 100%: A prospective agreement study [file sj-docx-1-dhj-10.1177_20552076231211169.docx]

# Supplementary Appendix 1

| **Table S1.** Demographic data of the 15 volunteers* | | | | | | |
| --- | --- | --- | --- | --- | --- | --- |
| Volunteer* | Age, years | Sex | Fitzpatrick scale | Height, m | Weight, kg | BMI, kg/m^2^ |
| 1 | 27 | male | 5 | 1.68 | 73 | 25.9 |
| 2 | 30 | male | 2 | 1.80 | 77 | 23.8 |
| 3 | 42 | male | 3 | 1.80 | 74 | 22.8 |
| 4 | 28 | male | 4 | 1.79 | 87 | 27.2 |
| 5 | 32 | female | 1 | 1.65 | 58 | 21.3 |
| 6 | 27 | male | 3 | 1.80 | 73 | 22.5 |
| 7 | 24^1^ | female | 1 | 1.65 | 54 | 19.8 |
| 8 | 28^1^ | male | 3 | 1.89 | 85 | 23.8 |
| 9 | 32 | male | 3 | 1.73 | 73 | 24.4 |
| 10 | 20 | female | 2 | 1.79 | 66 | 20.6 |
| 11 | 32^1^ | male | 2 | 1.80 | 70 | 21.6 |
| 12 | 26 | male | 2 | 1.80 | 83 | 25.6 |
| 13 | 21 | female | 2 | 1.66 | 60 | 21.8 |
| 14 | 29 | male | 4 | 1.85 | 80 | 23.4 |
| 15 | 19 | female | 2 | 1.70 | 63 | 21.8 |
| * In the order of study recruitment  ^1^ Volunteers 7, 8 and 11 were excluded: 7 due to feeling uncomfortable, 8 due to organisational reasons, 11 due to suspected Wolff-Parkinson-White Syndrome.  BMI, body mass index | | | | | | |

| **Table S2.** Individual agreements between oxygen saturation (SpO_2_) measured in-ear (c-med° alpha) and on the finger (Rad-5) of the twelve volunteers | | | | |
| --- | --- | --- | --- | --- |
| Volunteer* | Bias  (95% CI), % | Lower LOA  (95% CI), % | Upper LOA  (95% CI), % | A_rms_ |
| 1 | 0.5 | -1.6 | 2.6 | 1.2 |
| 2 | 0.5 | -1.8 | 2.5 | 1.2 |
| 3 | -0.0 | -2.1 | 2.1 | 1.1 |
| 4 | -0.7 | -4.6 | 3.1 | 2.1 |
| 5 | -0.1 | -6.0 | 5.8 | 3.0 |
| 6 | -1.0 | -3.7 | 1.6 | 1.7 |
| 9 | -0.1 | -6.1 | 5.9 | 3.0 |
| 10 | 0.7 | -0.9 | 2.3 | 1.1 |
| 12 | -1.1 | -3.2 | 1.1 | 1.5 |
| 13 | 1.9 | -1.2 | 5.0 | 2.5 |
| 14 | -1.2 | -3.1 | 0.7 | 1.6 |
| 15 | -0.5 | -2.8 | 1.7 | 1.3 |
| * Volunteers completed the study  To illustrate the small 95 % CIs, one digit after the decimal point is given, although the SpO_2_ is only measured as an integer.  CI = confidence interval, LoA = limit of agreement, A_rms_ = accuracy using root mean square error. | | | | |

| SpO_2_ difference [Rad-5 – c-med° alpha], % | 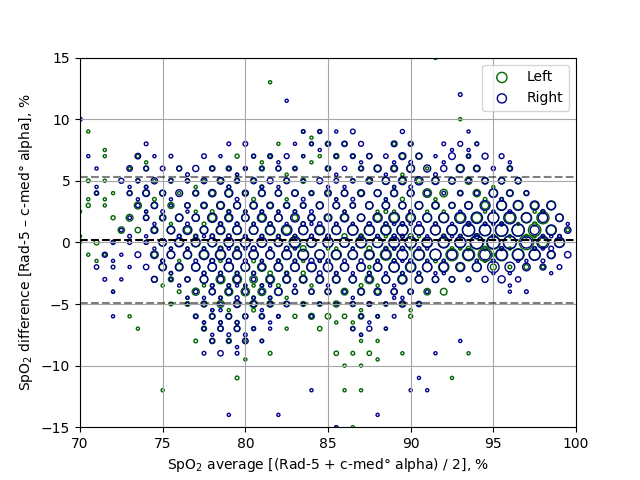 |
| --- | --- |
| SpO_2_ average [(Rad-5 + c-med° alpha) / 2], % | |
| **Supplementary Figure S1**. Bland Altmann analysis of the Rad-5 and the c-med alpha° of the SpO2 during the complete desaturation exercise. Differences between simultaneous SpO_2_ readings of the c-med° alpha in-ear oximetry and the finger pulse oximeter (Masimo Rad-5). In-ear values were taken in both ears; the SpO_2_ pairs of the fingers with the left ear are green, those with the right ear are blue. The thick dashed line indicates the mean difference of the measurements (bias: 0.2% [0.2% to 0.3%]), the thin dashed lines the 95% limits of agreement (lower level: -4.9% to -4.8%], upper level 5.3% [5.3% to 5.4%]). The size of markers is proportional to the number of readings. | |
